# Reusable surface amplified nanobiosensor for the sub PFU/mL level detection of airborne virus

**DOI:** 10.1038/s41598-021-96254-2

**Published:** 2021-08-18

**Authors:** Junghyun Shin, Hyeong Rae Kim, Pan Kee Bae, Haneul Yoo, Jeongsu Kim, Yoonji Choi, Aeyeon Kang, Wan S. Yun, Yong Beom Shin, Jungho Hwang, Seunghun Hong

**Affiliations:** 1 Department of Physics and Astronomy, Seoul National University, Seoul, 08826 Korea; 2 Department of Physics and Astronomy, and Institute of Applied Physics, Seoul National University, Seoul, 08826 Korea; 3 Gas Metrology Group, Korea Research Institute of Standards and Science (KRISS), Daejeon, 34113 Korea; 4 School of Mechanical Engineering, Yonsei University, Seoul, 03722 Korea; 5 BioNano Health Guard Research Center (H-GUARD), Daejeon, 34141 Korea; 6 Bionanotechnology Research Center, Korea Research Institute of Bioscience and Biotechnology 10 (KRIBB), Daejeon, 34141 Korea; 7 Department of Bioengineering, KRIBB School, University of Science and Technology (UST), Daejeon, 34141 Korea; 8 Department of Chemistry, Sungkyunkwan University, Suwon, 16419 Korea

**Keywords:** Biological techniques, Biophysics, Biotechnology

## Abstract

We developed a reusable surface-amplified nanobiosensor for monitoring airborne viruses with a sub-PFU/mL level detection limit. Here, sandwich structures consisted of magnetic particles functionalized with antibodies, target viruses, and alkaline phosphatases (ALPs) were formed, and they were magnetically concentrated on Ni patterns near an electrochemical sensor transducer. Then, the electrical signals from electrochemical markers generated by ALPs were measured with the sensor transducer, enabling highly-sensitive virus detection. The sandwich structures in the used sensor chip could be removed by applying an external magnetic field, and we could reuse the sensor transducer chip. As a proof of concepts, the repeated detection of airborne influenza virus using a single sensor chip was demonstrated with a detection limit down to a sub-PFU/mL level. Using a single reusable sensor transducer chip, the hemagglutinin (HA) of influenza A (H1N1) virus with different concentrations were measured down to 10 aM level. Importantly, our sensor chip exhibited reliable sensing signals even after more than 18 times of the repeated HA sensing measurements. Furthermore, airborne influenza viruses collected from the air could be measured down to 0.01 PFU/mL level. Interestingly, the detailed quantitative analysis of the measurement results revealed the degradation of HA proteins on the viruses after the air exposure. Considering the ultrasensitivity and reusability of our sensors, it can provide a powerful tool to help preventing epidemics by airborne pathogens in the future.

## Introduction

Respiratory viruses such as influenza and corona can spread out through the air and cause significant health problems. For example, influenza epidemics have been causing worldwide respiratory deaths ranging from 290,000 to 650,000 each year^1^. The outbreak of coronavirus disease-2019 (COVID-19) caused more than 1 million deaths worldwide^2^. Although vaccination can be an efficient strategy to reduce its hazardous impact, it often takes time to produce an effective vaccine.

Another strategy can be a real-time monitoring system which can detect and alarm respiratory viruses in the air before entering human bodies. Extensive efforts have been given to detect respiratory viruses in the air^3–11^. For example, several groups successfully trapped influenza virus in the air and measured its amount using a quantitative polymerase chain reaction (qPCR) method with a high sensitivity and selectivity^3–6^. However, the qPCR-based method takes some time to get a detection result, and it is not suitable for fast monitoring systems. In other works, nanobiosensors such as silicon nanowire-based biosensors have been utilized to detect influenza viruses captured from the air^8,9^. However, nanobiosensors are often very expensive. Furthermore, the detection limit of nanobiosensors is usually a few PFU level, which may not be enough to detect airborne viruses with a concentration of its threshold infectious dose in the air.

Herein, we report a reusable surface-amplified nanobiosensor for monitoring airborne viruses with a sub-PFU/mL level detection limit. In this method, antibody-functionalized magnetic particles were utilized to form sandwich structures with target viruses and alkaline phosphatase (ALP) enzyme, and they were trapped on the Ni patterns near electrochemical sensor transducers. Then, the electrochemical markers generated by trapped ALPs were measured by the underlying sensor for the highly-sensitive detection of the viruses. The used sandwich structures could be removed via an external magnetic field, enabling repeated sensing measurements. As a proof of concepts, we demonstrated the repeated detection of airborne influenza virus using a single sensor chip with a sub-PFU/mL level detection limit. Our sensor could be utilized repeatedly, for more than 18 times, to measure the hemagglutinin (HA) of influenza A (H1N1) virus, a widely-used target protein for detecting influenza virus^12–15^, with the concentration ranging from 10 aM to 1 nM. Importantly, our sensor chip provided consistent signals even after repeated sensing measurements over the concentration ranges. Moreover, we could utilize our sensor to measure airborne influenza viruses collected from the air with a detection limit down to 0.01 PFU/mL. Interestingly, the detailed quantitative analysis of sensor signals revealed the degradation of HA protein on the virus after the air exposure. Considering the ultra-sensitivity and reusability, our method can be a key stepping stone toward the continuous monitoring system of airborne pathogens, and it can provide a powerful tool for controlling epidemics by preventing airborne pathogens from entering a human body.

## Results and discussion

**Figure 1:**
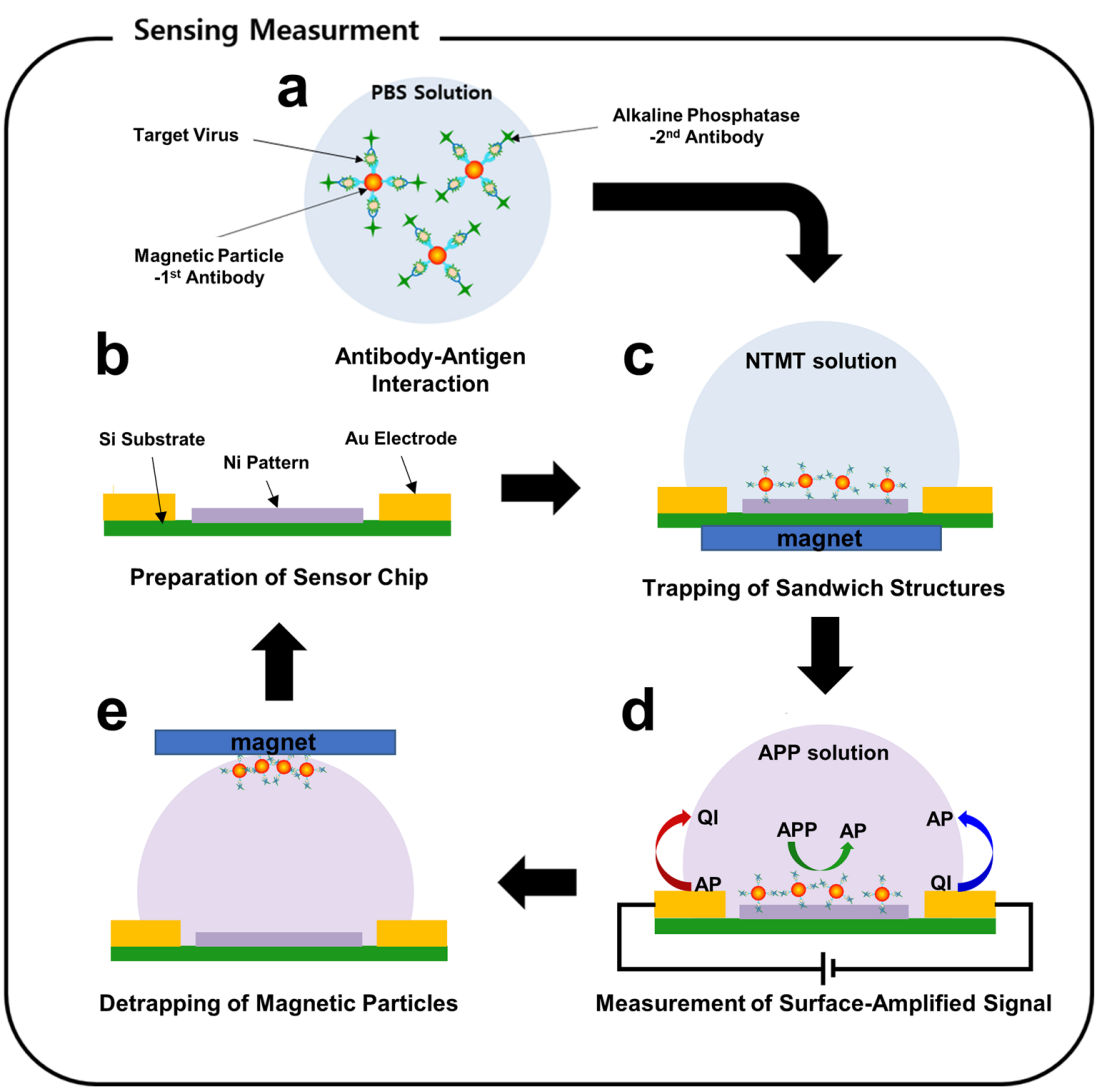
Schematic diagram depicting the cyclic process for the repeated sensing measurements of surface-amplified sandwich immunoassay. (**a**) Preparation of sandwich structures in PBS solution including magnetic particles conjugated with 1st antibody, alkaline phosphatase (ALP) conjugated with 2nd antibody, and influenza viruses. (**b**) Fabrication of a reusable sensor transducer chip including an interdigitated gap sensor and ferromagnetic Ni patterns. (**c**) Trapping of sandwich structures on Ni patterns between Au electrodes via an external magnetic field. (**d**) Electrochemical sensing measurement of surface-amplified electrical signals. (**e**) Detrapping of sandwich structures via an external magnetic field with an opposite direction to that for trapping.

Figure 1 shows a schematic diagram describing the repeated sensing process of our surface-amplified immunoassay method. Detailed processes are provided in the “Methods” section. Firstly, magnetic particles were functionalized with 1st antibody which selectively binds to HA protein on influenza A (H1N1) virus surface. The schematic diagram of our magnetic particles is presented in supplementary information (Fig. S1). Previous works showed that such magnetic particles maintain their magnetic properties even after the functionalization with biomaterials^16,17^. Alkaline phosphatase (ALP) enzyme was conjugated with its 2nd antibody. The magnetic particles and ALP were mixed with the target solution so that the antibodies bind to the target viruses, resulting in sandwich structures including magnetic particles, influenza virus, and ALP enzyme (Fig. 1a). The solution of the sandwich structures was placed on the fabricated sensor chip including two gold electrodes (90 nm Au on 10 nm Ti, 63 fingers of 10 μm width, 30 μm gap between the fingers, 2500 μm length) and an array of 125 × 150 ferromagnetic Ni patterns (10 nm Au on 90 nm Ni, 4 μm × 8 μm) in-between (Fig. 1b). We used Ni because it has been extensively utilized for practical applications due to some advantageous properties such as a high oxidation resistance and a low fabrication cost^18–20^. When an external magnetic field was applied, the Ni patterns were magnetized. Then, the patterns attracted magnetic particles in the solution. As a result, the sandwich structures including the influenza virus were concentrated on the ferromagnetic patterns in our sensors (Fig. 1c). 4-aminophenyl phosphate (APP) solution was added to the sandwich structure. Here, ALP enzyme on the Ni patterns generated aminophenols (APs), electrochemical markers, which were measured by using interdigitated Au electrodes with the bias voltages of + 0.1 V and – 0.1 V (Fig. 1d). In this method, since a large number of marker molecules were generated by one enzyme molecule in a sandwich structure with only a single virus and many sandwich structures were concentrated on the sensor surface, we could achieve highly-amplified sensor signals. After the sensing process, the used sandwich structures were removed from the sensor chip by applying an external magnetic field in an opposite direction (Fig. 1e). This method allows us to measure extremely low concentration virus solution via signal amplification near the sensor surfaces and to use a single electrode chip for repeated sensing measurements.

**Figure 2:**
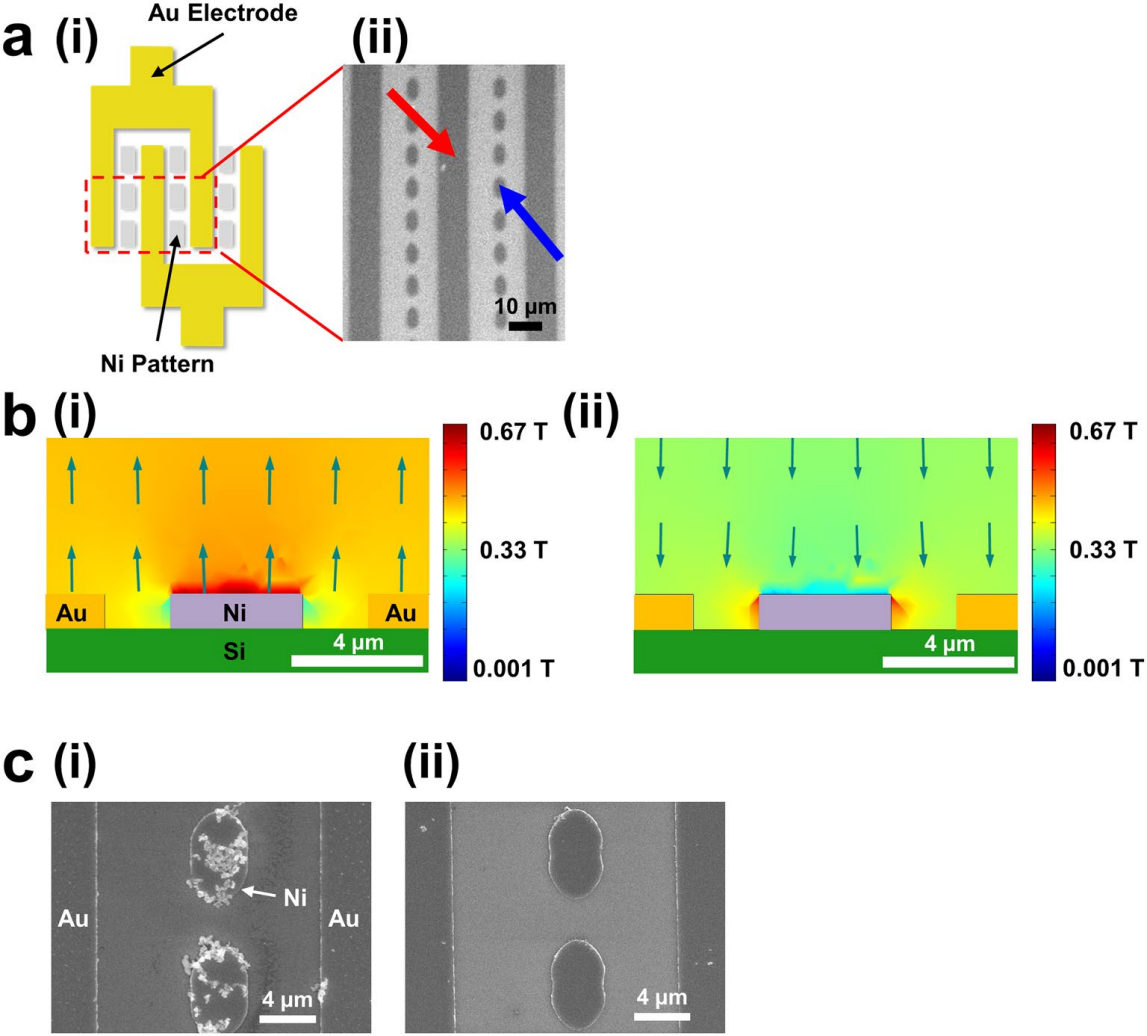
Basic trapping and detrapping operations of our reusable sensor chip. (**a**) Schematic diagram (**i**) showing our reusable sensor chip and the field emission scanning electron microscopy (SEM) image (**ii**) of a sensor chip. (**b**) Simulation results showing cross-sectional views of a magnetic field strength around a Ni pattern during trapping (**i**) and detrapping (**ii**) processes. (**c**) SEM images after trapping (**i**) and detrapping (**ii**) magnetic particles on Ni patterns.

Figure 2a shows a schematic diagram (i) and a scanning electron microscopy (SEM) image (ii) of our reusable interdigitated gap sensor chip. In the SEM image, an *Au electrode* and *Ni patterns* are marked with *red* and *blue* arrows, respectively (Fig. 2a(ii)). The image shows that Ni pattern arrays (4 μm × 8 μm) were fabricated uniformly between interdigitated Au electrodes. Thus, we can expect that the ferromagnetic patterns could evenly deflect external magnetic fields around the electrode structures so that magnetic particles could be trapped on the patterns without significant aggregations.

Figure 2b depicts simulation results showing cross-sectional views of a magnetic field strength around a Ni pattern during trapping (i) and detrapping (ii) processes. The direction and size of each arrow represent those of the magnetic field on that location. For the trapping process, 450 mT of an external magnetic field in an upward direction was applied to a Ni pattern between Au electrodes (Fig. 2b(i)). In this case, the Ni pattern was magnetized along the direction of the field. Due to the magnetic field from the magnetized Ni pattern, a magnetic field strength near the Ni pattern was stronger than those in other regions. For example, the simulation results show that magnetic fields right above the Ni patterns were approximately 600 mT, while those of other regions were approximately 450 mT (Fig. 2b(i)). Since magnetic particles, due to their magnetic dipole moment, tended to be placed on the strong magnetic field region, they were attracted to the top of Ni patterns and trapped there during a trapping process. Also, note that the strong magnetic field region extended only slightly above the Ni pattern (Fig. 2b). Thus, we can expect that only a thin layer of magnetic particles could be trapped on the Ni patterns without a formation of thick aggregations^18,20^.

For the detrapping process, a rather weak magnetic field (350 mT) in a downward direction opposite to the previously-applied magnetic field was applied to the Ni pattern (Fig. 2b(ii)). However, due to the hysteresis of ferromagnetic Ni patterns, the magnetic polarization of the Ni pattern remained in an upward direction. Since the magnetic fields by the remaining magnetic polarization of the Ni patterns had an opposite direction to that of the external field, the magnetic field strength just above the Ni pattern should be lower than those in other regions. Our simulation results show that the regions above the Ni pattern are darker than other regions, indicating weak magnetic fields. In this case, we can expect that the magnetic particles trapped on the Ni patterns were released from the pattern surface.

Figure 2c *(i)* and *(ii)* show the SEM images of Ni patterns after *trapping* and *detrapping* of magnetic particles, respectively. Bright particles represented the magnetic particles. The detailed experimental procedure is provided in the “Methods” section. In the trapping process, an external magnetic field of 450 mT was applied normal to the sensor surface, resulting in the selective trapping of magnetic particles only on the Ni patterns (Fig. 2c(i)). Moreover, the trapped particles formed a single layer without forming a large aggregation of magnetic particles.

On the other hand, when a rather weak magnetic field (350 mT) was applied in an opposite direction, magnetic particles were completely removed and, thus, there were no magnetic particles on the Ni patterns (Fig. 2c(ii)). These results show that magnetic nanostructures such as sandwich structures including magnetic particles could be trapped or detrapped on the Ni patterns in our sensor structures, simply by controlling external magnetic fields.

**Figure 3:**
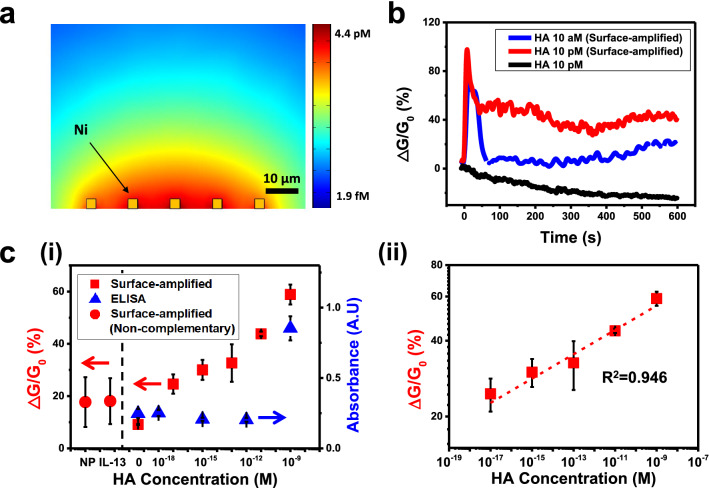
Repeated sensing measurements to detect hemagglutinin (HA) proteins of influenza A (H1N1) virus via surface-amplified sandwich immunoassay. (**a**) Simulation result showing AP concentrations after AP diffusion in the surface-amplified sandwich immunoassay. (**b**) Real-time sensor responses to various concentrations of HA with and without the surface amplification of signals. (**c**) Dose-dependence response curves of the surface-amplified immunoassay (n = 3) and conventional ELISA measurements (n = 5) using the same 1st and 2nd antibodies **(i)**. Each data point and error bar represents the average value and standard deviation of the experiments. Log–log scale plot showing linearity of our sensor response against HA concentrations (**ii**).

When sandwich structures including magnetic particles and enzymes were trapped on Ni patterns, APP solution was applied so that the enzyme generates 4-Aminophenol (AP) (Fig. 1d). Here, the generated AP, as a electrochemical marker, induced the electrical currents between two electrodes in our sensor surface, which were used as a signal of our sensor (Fig. 3). In this case, the concentration distribution of the generated AP near the sensor surface determines the sensor signals. Figure 3a depicts the cross-sectional view of numerical simulation results about the concentration distribution of AP generated by the enzyme in the sandwich structures on the Ni patterns. The yellow rectangles at the bottom of the image represent the Ni patterns. Other regions of the image show the concentration of simulated APs in the buffer solution. In the simulation, 39 μL of buffer solution was assumed in a cylinder type well which has a 2.5 mm radius and 2 mm height. The 5 × 20 array of Ni patterns (4 μm × 8 μm) was placed at the bottom of the well and used to generate APs. We also assumed that the AP was continuously generated on the surfaces of the Ni pattern array and diffused into the buffer solution for 600 s. In the simulation results, the regions *near* or *far from* the Ni patterns appeared *red* or *light blue*, respectively, indicating that AP concentrations near the Ni patterns were higher than those in regions far from them (Fig. 3a). Note that the AP concentration within 3 μm away from the Ni pattern array is 6,400 times higher than the averaged concentration of the whole solution, implying a rather large sensor signal. The simulation results indicate that, in our sensors, AP molecules were generated by the enzyme concentrated near our sensor surface, which enabled large sensor signals and the detection of target molecules with an extremely-high sensitivity.

Figure 3b shows typical real-time data of the sensing measurements using hemagglutinin (HA) protein as a target. HA is the protein on influenza A (H1N1) virus surface and is widely used as a target protein to detect the influenza virus^12–15^. The detailed experimental procedure is presented in the “Methods” section. Briefly, two different solutions of sandwich structures (comprised of magnetic particles, HAs, and ALPs) were prepared using HA solutions with different concentrations of 10 aM and 10 pM. Then, HA sensing experiments were performed using the solution with (red or blue lines) or without (black line) trapping the sandwich structures on the Ni patterns of our sensor. Here, the relative conductance change (ΔG⁄G_0_) of the sensor chip was used as a sensor signal, where *ΔG* and *G*_*0*_ are the *conductance change* and *original conductance* of the sensor chip, respectively. For all experiments in the graph, a single sensor chip was utilized repeatedly. When APP solution was applied to the sandwich structures trapped on the sensor surface, the sensor signal increased sharply and decreased back to a stabilized value at around 600 s (red and blue lines). The results can be attributed to the redox reaction with aminophenol (AP) and quinone imine (QI) near the cathode and anode electrodes of our sensor. When APP solution was first injected, ALP enzyme in the sandwich structures hydrolyzed most of APP near the sensor surface quickly and generated AP. The generated AP near the cathode electrode were immediately converted to QI by oxidation reactions and provided electrical currents in the electrode, resulting in a sharp increase of sensing signals as reported previously^21–23^. However, after a short time period, most of AP generated from APP was converted to QI, and the sensor signals began to decrease^24^. In this case, the generated QIs diffused to the anode electrode and were converted back to AP by reduction processes. The reduced AP on the anode electrode could diffuse back to the cathode and oxidized again to provide electrical currents^25^. After a while, such oxidation and reduction processes were repeated on cathode and anode electrodes and reached an equilibrium condition, resulting in stable current signals^26^. On the other hand, when the sandwich structures were dispersed in solution without being trapped on the sensor surface, the injected APP was converted to AP slowly over the entire solution. Thus, we could not observe the immediate increase of current signals, and it usually took a rather long time (much longer than 600 s) to reach an equilibrium signal (black line in Fig. 3b). Since the signals with trapped sandwich structures were stabilized around 600 s after the APP injection, the saturated sensor signals were utilized as a sensor signal. Note that the sensor signal for the 10 aM HA solution (blue line) was much smaller than that for the 10 pM HA solution (red line), indicating that our method can be utilized to quantitatively measure the concentration of target solutions. Also, it should be mentioned that the sensor signal without trapping sandwich structures from 10 pM HA solution (black line) was even smaller than that with trapped sandwich structures from 10 aM solution (blue line), indicating the signal enhancement effect by more than 10^6^ times. Presumably, the enzyme in the sandwich structures trapped near the electrodes generated AP with a high concentration near our sensor surface, enhancing the sensor signals. These results show that our strategy of trapping the enzyme-functionalized sandwich structures on the sensor surface can significantly improve the sensor sensitivity.

Figure 3c (i) shows the dose-dependent responses of the HA sensing measurements with our surface-amplified immunoassay strategy (red squares, n = 3) as well as conventional enzyme-linked immunosorbent assay (ELISA) methods (blue triangles, n = 5). Here, HA solutions with their concentrations ranging from 10 aM to 1 nM were used for the surface-amplified immunoassay. Also, non-specific responses of our sensor were tested by measuring the sensor responses to non-targeted nucleoproteins (NP) of influenza A (H1N1) virus and interleukin 13 (IL-13) with a 10 pM concentration (red circles). It should be noted that all 24 sensing measurements of our surface amplified immunoassay were performed on a single sensor chip (Fig. 3c(i)). Conventional ELISA measurements were performed using the same antibody and HA target protein as our sensors. The detailed experimental procedures are presented in supplementary information. Our sensor began to show responses to 10 aM HA solution, and the responses increased with increasing HA concentrations. However, the non-specific responses to NP and IL-13 were negligibly small compared to those to HA sensing results. These results clearly show the high sensitivity and selectivity of our surface amplified immunoassay. On the other hand, the conventional ELISA method using the same antibody shows sensing signals from the 1 nM HA solution. Although the same antibodies with their binding constants of a few nanomolar range were used in both sensing measurements, our surface amplified immunoassay showed a detection limit which is lower, by more than ~ 10^7^ times, than that of the conventional ELISA. Presumably, the signal amplification by enzyme near the sensor surface should have enabled such signal enhancements as discussed in Fig. 3b. Importantly, in this sensing experiment, a single sensor was utilized to perform more than 20 times of repeated measurements, while still providing consistent results at each concentration of HA (red dots and squares in Fig. 3c(i)). The results show that our sensor can be utilized repeatedly for the detection of hazardous pathogens with a high sensitivity and selectivity.

For a detailed analysis about our sensor responses, we performed a numerical simulation and a fitting for the measured sensor signals (Fig. 3c(ii)). Previous works show that sandwich structure-based biosensors like our sensors exhibited a linear response for different target concentrations^27–31^. Also, a numerical analysis was performed to confirm the linear responses of our sensor. Here, we first set-up differential rate equations regarding individual antibody-antigen bindings during the formation process of sandwich structures and performed a numerical simulation to calculate the sensor signals for different target concentrations (Fig. S2 in supplementary information). The results show that our sensor based on sandwich structures should exhibit a linear response at a rather low target concentration condition. Accordingly, the measured sensor response data were fitted by a linear fitting curve via the linear regression analysis, showing a good fitting result with R^2^ = 0.946 (red dashed line in Fig. 3c(ii)). These results show that our sensors can be used to measure a target concentration in a quantitative manner. Furthermore, it also should be mentioned that the sensor exhibited a linear response over a very wide range of target concentrations of 10 aM ~ 1 nM. For various practical applications, a wide analytical range is usually advantageous. However, such a wide analytical range can also limit the resolution of a sensor, which can be disadvantageous for some applications requiring a precise quantitative analysis. For example, a two-fold increase of target concentrations can change our sensor signals only by ~ 25%. Similar behavior has been reported previously for other biosensors based on antibody bindings^32,33^.

**Figure 4:**
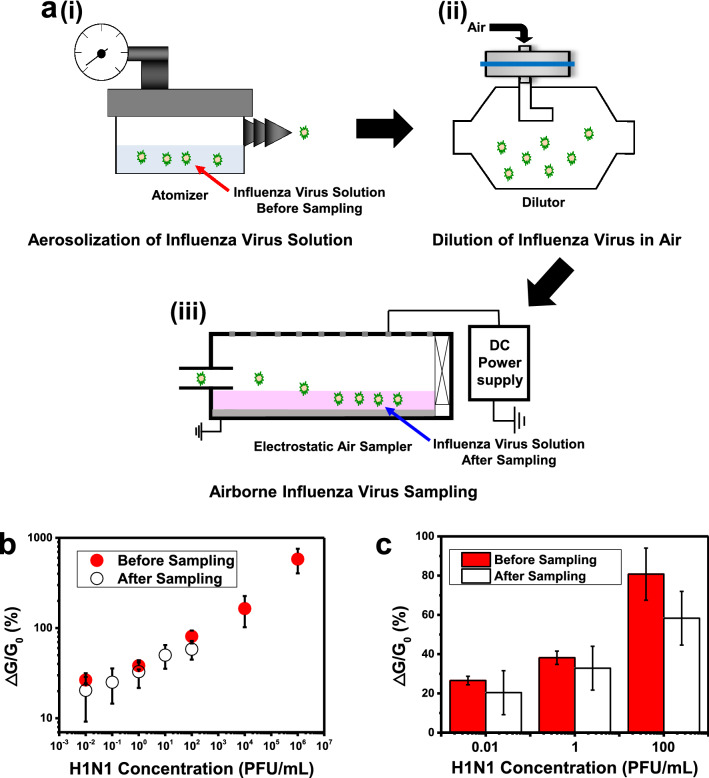
Repeated sensing measurements for the detection of airborne influenza A (H1N1) virus via the surface-amplified sandwich immunoassay. (**a**) Schematic diagram depicting the aerosolization and collection of influenza virus solution. (**i**) Aerosolization of influenza virus solution. (**ii**) Dilution of the aerosolized influenza virus with clean air. (**iii**) Collection of the aerosolized influenza virus. (**b**) Dose-dependence response curves of surface-amplified immunoassay to influenza viruses before and after the aerosolization and collection processes. Each data point and error bar represents the average value and standard deviation of the experiments (n = 3). (**c**) Comparison of the sensing measurement results obtained before and after the sampling processes.

As a proof of concept, our sensor was utilized for the detection of airborne influenza viruses (Fig. 4). Figure 4a shows the schematic diagrams of the experimental set-ups for the aerosolization and collection of influenza viruses. In an atomizer, a solution of influenza viruses was released into the air as aerosols with a flow rate of 2 L/min (Fig. 4a(i)). Then, the aerosolized viruses were diluted with clean air to 100 L/min in a dilutor (Fig. 4a(ii)). Finally, a lab-made electrostatic air sampler was used for sampling the airborne influenza viruses (Fig. 4a(iii)). In the air sampler, the airborne viruses were charged by air ions generated from the tungsten electrode wires via corona discharge. The charged viruses were attracted toward the ground electrode covered with PBS solution, and they were captured in the PBS solution^34^. The number of collected viruses in the solution could be estimated via a conventional analysis method based on a particle counter as shown in the “Methods” section. The solutions of collected viruses were used as a target solution for the sensing measurement of our sensors without any further treatments.

Figure 4b displays the dose-dependent responses of our surface-amplified immunoassay using the target solutions of influenza virus before (hollow) and after (red) the aerosolization and collection processes described in Fig. 4a. Here, a single sensor chip was utilized to perform all sensing measurements for both virus solutions with different concentrations ranging from 10^–2^ PFU/mL to 10^6^ PFU/mL. The same set of measurements were performed repeatedly by three sensor chips to obtain error bars for the data. For both viruses before and after the aerosolization step, our sensor began to respond to virus solutions with its concentration of 0.01 PFU/mL level. The responses also increased linearly as the concentrations of the virus solutions increased. These results clearly show that our sensor can reliably detect airborne influenza viruses with an sub-PFU/mL level sensitivity using a single sensor chip. Considering that conventional immunoassays usually show a detection limit over a few PFU/mL, our method can be a major breakthrough, and it can be utilized for versatile practical applications such as medical screening and biological research^35,36^.

It is also very interesting to note that, even with the same concentrations, the solutions with viruses after an aerosolization step exhibited a sensor signal slightly smaller than those before the step. Figure 4c shows a more detailed comparison of the dose-dependence responses for influenza virus solutions before and after the sampling processes. The sensing results at the concentration of 0.01, 1, and 100 PFU/mL were statistically analyzed. The signals from the collected airborne influenza solutions (hollow bars) were, on average, 21.6% lower than those of the same concentration virus solutions before sampling (red bars). One plausible explanation can be the reduced binding activity of HA protein on the viruses after the aerosolization step. In our experiment, the concentrations of virus solutions were measured by estimating the number of viruses in the solution as shown in the “Methods” section, while our sensor signals rely on the binding activities of the HA proteins as well as the number of viruses. The slightly reduced sensor signals imply that the binding activity of viruses after the aerosolization was reduced, possibly due to the structural deformation of HA proteins on the virus surfaces during the aerosolization and collection processes. This result about a quantitative measurement shows that our method can be a useful tool for basic researches as well as practical applications.

## Conclusions

A reusable surface-amplified nanobiosensor was developed for monitoring airborne viruses with a sub-PFU/mL level detection limit. In this strategy, magnetic particles functionalized with antibody molecules were used to form sandwich structures with target viruses and ALP enzyme, and they were magnetically collected on Ni patterns close to an electrochemical sensor transducer. Then, electrochemical markers generated by ALP enzyme were measured via the electrochemical sensor transducer for the highly-sensitive detection of viruses. The used sensor transducer chip could be reused after removing the trapped sandwich structures via an eternal magnetic field. As a proof of concepts, our sensor chip was utilized to detect airborne influenza virus with a concentration down to a sub-PFU/mL level. A single reusable sensor chip was used repeatedly to measure HA target proteins of influenza A (H1N1) virus down to 10 aM level. Even after 18 times of repeated sensing measurements, the sensor chip showed consistent results. Additionally, influenza virus solutions with different concentrations could be measured down to 0.01 PFU/mL level. Importantly, a quantitative analysis of our sensor signals revealed the degradation of HA proteins on the viruses after the air exposure. Considering the reusability and ultrasensitivity, our method could be a powerful tool for the monitoring of airborne pathogens and help to prevent epidemics by airborne pathogens.

## Methods

### Material

Amine coated superparamagnetic magnetic particles (SiMAG-Amine, with its hydrodynamic diameter of 1 µm, 50 mg/mL in phosphate buffer saline (PBS) solution) were purchased from Chemicell GmbH (Germany). Hemagglutinin (HA, A/California/06/2009(H1N1)), anti-HA monoclonal 1st antibody, HRP conjugated anti-HA monoclonal 1st antibody, and anti-HA monoclonal 2nd antibody were obtained from Immune Technology Corp (USA). Influenza A virus solutions (A/California/07/2009 pdmH1N1) were donated by BioNano Health Guard Research Center (H-GUARD).

### Fabrication of reusable sensor transducer chips including interdigitated electrodes and ferromagnetic nickel patterns

Interdigitated Au gold electrodes (90 nm Au on 10 nm Ti, Ti/Au) and ferromagnetic Ni patterns (10 nm Au on 90 nm Ni, Ni/Au patterns of size 4 µm × 8 µm) were fabricated on a SiO_2_ substrate via conventional photolithography processes.

### Trapping and detrapping of magnetic particles on a reusable sensor transducer

Amine coated superparamagnetic magnetic particle solution was diluted to a concentration of 600 µg/mL in PBS solution. Using a pipet, the 20 µL droplet of the solution was placed on the sensor transducer chip surface. The 450 mT of an external magnetic field was applied for 1 min 30 s to the sensor chip for trapping the magnetic particles on the Ni patterns. After the trapping process, magnetic particles non-specifically trapped on the surface regions without Ni patterns were removed by pipetting the solution away. For the detrapping of the trapped magnetic particles, the 350 mT of an external magnetic field with an opposite direction was first applied for 2 min to the sensor transducer chip in solution environments. Then, the sensor transducer chip was washed by acetone, ethanol, deionized (DI) water for removing any remaining magnetic particles.

### Preparation of magnetic particles functionalized with 1st antibody

Firstly, the anti-HA monoclonal 1st antibody solution was diluted to a 200 nM concentration in 150 µL PBS solution. 18 µL of Amine coated superparamagnetic magnetic particle solution was prepared. By using a magnetic separator, the magnetic particles were washed 2 times with 200 µL of 2-(N-morpholino)ethanesulfonic acid (MES) buffer (0.1 M, pH 6.0). The magnetic particles were separated from the solution by a magnet. After removing the supernatant, 300 µL MES buffer was added to the magnetic particles. 1-ethyl-3-(3-dimethylaminopropyl)carbodiimide (EDC) and N-hydroxysuccinimide (NHS) (GE Healthcare, USA) were dissolved in MES buffer. 240 µL of the magnetic particle solution was mixed with 60 µL of the MES buffer containing 5 mM EDC and 20 mM NHS. 150 µL of the solution was added to the 1st antibody solution. The solution was gently mixed for 2 h at room temperature. The magnetic particles functionalized with 1st antibody were washed 3 times in 200 µL of PBS buffer by using the magnetic separator. The magnetic particles were separated from the solution by a magnet. After removing the supernatant, 300 µL PBS buffer was added to the magnetic particles.

### Preparation of ALP functionalized with 2nd antibody

An alkaline phosphatase labeling kit was purchased from dojindo molecular technologies (USA). 2nd antibody was conjugated with ALP following the technical manual provided by the vendor.

### Sandwich structures including HA protein

27 g of blocking reagent for ELISA (Roche, Swiss) was dissolved in 1 L of PBS buffer. HA protein solution was prepared with the desired concentration in the blocking buffer. 50 µL of the prepared magnetic particles functionalized with 1st antibody solution was added to 500 µL of the prepared HA solutions. The mixed solution was incubated at 37 °C for 2 h. The solution of ALP functionalized with 2nd antibody was diluted to 120 pM of 2nd antibody concentration in the blocking buffer. Then, 50 µL of the solution was injected into the mixed solution. The solution was incubated at 37 °C for 1 h. By using a magnetic separator, the sandwich structure solution was washed 3 times with 200 µL of PBS solution. The sandwich structures were separated from the solution by a magnet. After removing the supernatant, 100 µL of alkaline phosphatase buffer (NTMT) buffer (100 mM NaCl, 100 mM Tris–Cl (pH 9.0), 50 mM MgCl_2_, 1% tween 20) was added to the sandwich structures.

### Influenza virus aerosolization and collection

The detailed experimental setup is presented in Fig. S3. Influenza A (H1N1) virus solution was prepared following procedures in supplementary information. The stocks of influenza viruses were diluted with deionized water to 4.33 × 10^5^ PFU/mL. The solution was aerosolized using an atomizer (9302, TSI., USA) with a 2 L/min flow rate of clean compressed air. A diffusion dryer was used to eliminate moisture of the aerosolized virus solution. Then, the virus particles passed through a neutralizer (Soft X-ray charger 4530, HTC, Korea) to induce a Boltzmann charge distribution. The virus-laden air flow was diluted with clean air to 100 L/min and then placed into the air sampler. Airborne viruses entering the air sampler were captured on the ground electrode covered with PBS^34^.

### Quantification of airborne influenza virus collected after aerosolization

The collection efficiency (*η*_collection_) of the air sampler for the test viruses was calculated by the following equation:

1 $$\eta_{{{\text{collection}}}} = 1 - \frac{{{\text{C}}_{{{\text{on}}}}}}{{\text{C}}_{{{\text{off}}}}}$$

where *C*_*on*_ and *C*_*off*_ are the number concentrations of aerosolized viruses measured downstream of the sampler with a scanning mobility particle sizer (SMPS, TSI, USA) when the power is *on* and *off*, respectively. The virus collection efficiency $\left( {\eta_{{{\text{collection}}}} } \right)$ of the sampler was 0.7 ± 0.04 with the sampling flow rate of 100 L/min and the applied voltage of − 10 kV (Fig. S4 in supplementary information). The virus sampling was carried out for 60 min with an airflow rate of 100 L/min and an applied voltage of − 10 kV. The concentration of the collected virus sample (C_sample_) was calculated using the following equation:

2 $${\text{C}}_{{{\text{sample}}}} = \frac{{{\text{C}}_{{{\text{air}}}} {\text{*Q*}}\eta_{{{\text{collection}}}} {\text{*t}}}}{{\text{V}}}$$

where C_air_ is aerosolized virus concentration (4.33 × 10^3^ PFU/L), Q is air flow rate of the sampler (100 L/min), $\eta_{{{\text{collection}}}}$ is the collection efficiency (0.7), t is virus sampling time (60 min), and V is volume of collection medium (20 mL). Therefore, the concentration of collected virus sample was 910 PFU/mL.

### Sandwich structures including influenza viruses

Influenza virus solution with the desired concentration was prepared in the blocking buffer. Sandwich structures were prepared in the same way as in the preparation of sandwich structures including HA, except that influenza virus solution was used for forming sandwich structures instead of HA protein solution.

### Repeated sensing operations of reusable interdigitated gap sensors

A cylindrical Polydimethylsiloxane (PDMS, Sylgard™ 184, Sigma-Aldrich) well (5 mm diameter, 5 mm height) was placed on a reusable interdigitated gap sensor. 20 µL of 1% w/v bovine serum albumin (BSA) solution was introduced into the PDMS well. After 5 min, the BSA solution was removed. Then, the 20 µL of sandwich structure solution was placed in the well. The 450 mT of an external magnetic field was applied for 1 min 30 s to the sensor chip for trapping the sandwich structures on the Ni patterns. After the trapping process, the sandwich structures trapped on the sensor surfaces without Ni patterns were removed by pipetting the solution away. 0.2 V of bias voltage was applied to the Au electrodes of the sensor chip. The current of the sensor chip was measured with a bipotentiostat (µStat 400, DropSens). After the stabilization of the current signals, the stabilized current signals were recorded as baseline signals. Then, 20 µL of 10 mM APP solution in NTMT buffer was added to the sandwich structure solution. After 10 min, the current signals were recorded as sensing signals. When a sensing measurement was completed, the 350 mT of an external magnetic field was applied for 2 min to the sensor chip for detrapping the sandwich structures. The sensor chip was washed with acetone, ethanol, and DI water for removing the remained magnetic particles. The sensor chip was irradiated with ultraviolet (UV) light (278 nm wavelength) for UV/ozone surface cleaning.

## Supplementary Information

Supplementary Information.
