# Author Correction: Reusable surface amplified nanobiosensor for the sub PFU/mL level detection of airborne virus

**DOI:** 10.1038/s41598-021-98207-1

**Published:** 2021-09-15

**Authors:** Junghyun Shin, Hyeong Rae Kim, Pan Kee Bae, Haneul Yoo, Jeongsu Kim, Yoonji Choi, Aeyeon Kang, Wan S. Yun, Yong Beom Shin, Jungho Hwang, Seunghun Hong

**Affiliations:** 1 Department of Physics and Astronomy, Seoul National University, Seoul, 08826 Korea; 2 Department of Physics and Astronomy, and Institute of Applied Physics, Seoul National University, Seoul, 08826 Korea; 3 Gas Metrology Group, Korea Research Institute of Standards and Science (KRISS), Daejeon, 34113 Korea; 4 School of Mechanical Engineering, Yonsei University, Seoul, 03722 Korea; 5 BioNano Health Guard Research Center (H-GUARD), Daejeon, 34141 Korea; 6 Bionanotechnology Research Center, Korea Research Institute of Bioscience and Biotechnology 10 (KRIBB), Daejeon, 34141 Korea; 7 Department of Bioengineering, KRIBB School, University of Science and Technology (UST), Daejeon, 34141 Korea; 8 Department of Chemistry, Sungkyunkwan University, Suwon, 16419 Korea

Correction to: *Scientific Reports* 10.1038/s41598-021-96254-2, published online 18 August 2021

Aeyeon Kang and Wan S. Yun were omitted from the author list in the original version of this Article.

The Author Contributions section now reads:

“The key idea was conceived by J.H., S.H., A.K., and W.S.Y. J.S. designed the study, performed most experiments, analyzed the data, and wrote the manuscript. H.R.K. aerosolized and collected the virus. P.K.B. prepared the influenza virus solution. H.Y., J.K., and Y.C. wrote the paper. Y.S. prepared the influenza virus solution. J.H. aerosolized and collected the virus. S.H. is responsible for the project and contributed to data analyses. The manuscript was written through the contributions of all authors. All authors have given approval to the final version of the manuscript.”

The original Article has been corrected.

