# Supplementary material for: Reusable surface amplified nanobiosensor for the sub PFU/mL level detection of airborne virus
Source: Sci Rep. 2021 Aug 18;11:16776. doi: 10.1038/s41598-021-96254-2 (PMC8373909; doi:10.1038/s41598-021-96254-2)
Supplement: Supplementary file 1 — Supplementary Information. [file 41598_2021_96254_MOESM1_ESM.docx]

**Supplementary Information**

**Reusable Surface Amplified Nanobiosensor
for the Sub PFU/mL Level Detection
of Airborne Virus**

Junghyun Shin^a^, Hyeong Rae Kim^c^, Pan Kee Bae^e^, Haneul Yoo^a^, Jeongsu Kim^a^, Yoonji Choi^a^, Aeyeon Kang^h^, Wan Soo Yun^h^, Yong Beom Shin^e,f,g^, Jungho Hwang^d^, and Seunghun Hong^b^*

^a^Department of Physics and Astronomy, Seoul National University, Seoul 08826, Korea

^b^Department of Physics and Astronomy, and Institute of Applied Physics, Seoul National University, Seoul 08826, Korea

^c^Gas Metrology Group, Korea Research Institute of Standards and Science (KRISS), Daejeon 34113, Korea

^d^School of Mechanical Engineering, Yonsei University, Seoul 03722, Korea

^e^BioNano Health Guard Research Center (H-GUARD), Daejeon 34141, Korea

^f^Bionanotechnology Research Center, Korea Research Institute of Bioscience and Biotechnology 10 (KRIBB), Daejeon 34141, Korea

^g^Department of bioengineering, KRIBB School, University of Science and Technology (UST), Daejeon 34141, Korea

^h^Department of Chemistry, Sungkyunkwan University, Suwon 16419, Korea

**Fig. S1**

**
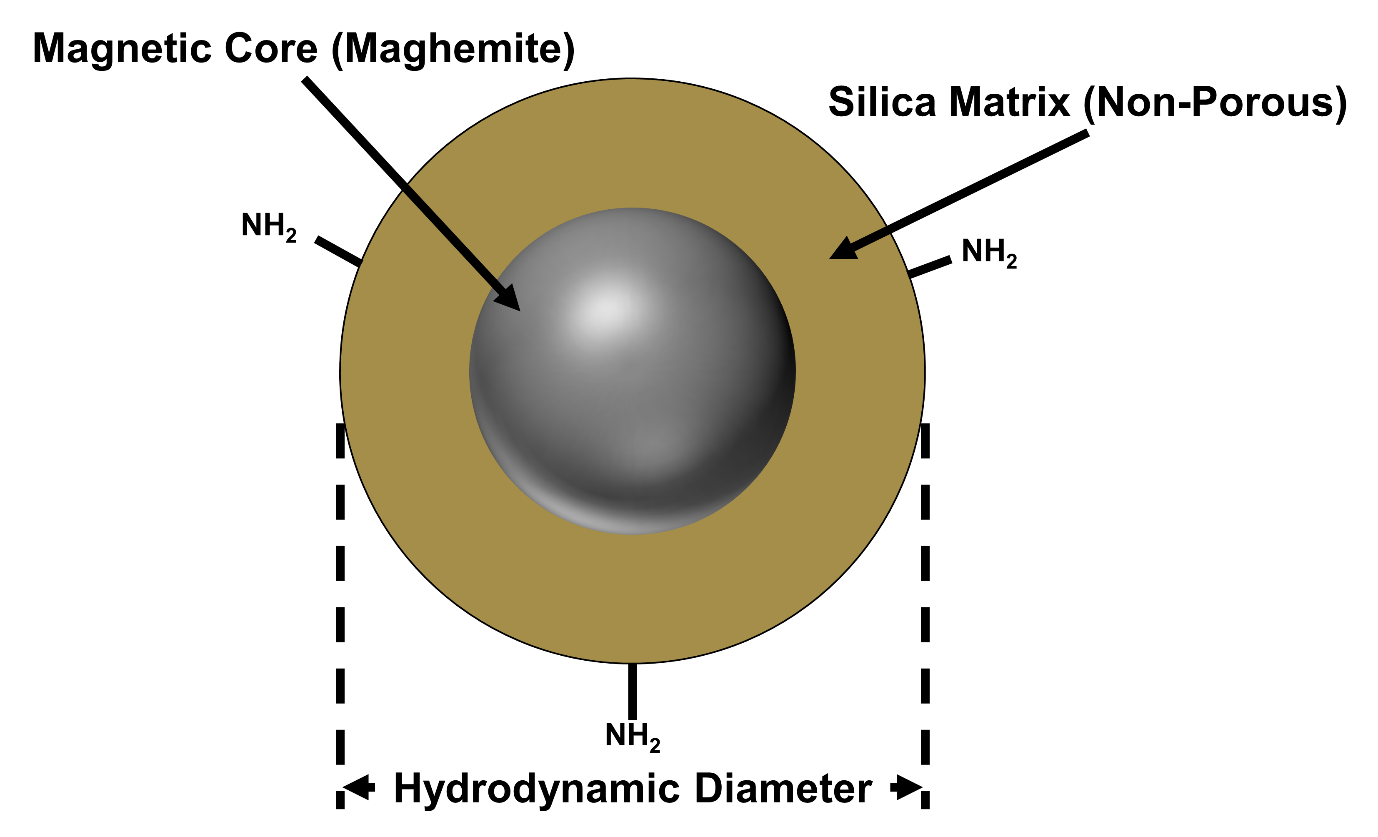
**

**Fig. S1** A schematic diagram of our magnetic particle. Magnetic particles consist of a magnetic core with a coating matrix of polymers and amino groups on the surface of them. The hydrodynamic diameter is ~ 1 μm.

**Fig. S2**

**Fig. S2** Numerical simulation results showing the concentration of sandwich structures ([ABC]) at different target concentrations ([B]). The results were obtained via the numerical simulation as following.

In this simulation, we first set up differential rate equations for the bindings between an antigen and an antibody during the formation process of a sandwich structure like

$\frac{d[A]}{dt}=-k_{a1}\left[ A \right]\left[ B \right]+k_{d1}\left[ AB \right]-k_{a1}\left[ A \right]\left[ BC \right]+k_{d1}\left[ ABC \right]$ (1)

$\frac{d[B]}{dt}=-k_{a1}\left[ A \right]\left[ B \right]+k_{d1}\left[ AB \right]-k_{a2}\left[ B \right]\left[ C \right]+k_{d2}\left[ BC \right]$ (2)

$\frac{d[C]}{dt}=-k_{a2}\left[ B \right]\left[ C \right]+k_{d2}\left[ BC \right]-k_{a2}\left[ AB \right]\left[ C \right]+k_{d2}\left[ ABC \right]$ (3)

$\frac{d[AB]}{dt}=k_{a1}\left[ A \right]\left[ B \right]-k_{d1}\left[ AB \right]-k_{a2}\left[ AB \right]\left[ C \right]+k_{d2}\left[ ABC \right]$ (4)

$\frac{d[BC]}{dt}=k_{a2}\left[ B \right]\left[ C \right]-k_{d2}\left[ BC \right]-k_{a1}\left[ A \right]\left[ BC \right]+k_{d1}\left[ ABC \right]$ (5)

$\frac{d[ABC]}{dt}=k_{a1}\left[ A \right]\left[ BC \right]-k_{d1}\left[ ABC \right]+k_{a2}\left[ AB \right]\left[ C \right]+k_{d2}\left[ ABC \right]$ (6)

where *[A], [B], and [C]* represent the concentrations of reaction components like *1^st^ antibody*, *antigen*, and *2^nd^ antibody*, respectively. The composite molecules comprised of different components are written like AB, BC, and ABC. Since the 1st and 2nd antibodies do not bind to each other, the component AC is excluded. $k_{a1}$ and $k_{d1}$ are an *association* and a *dissociation* rate constant between A and B, respectively. $k_{a2}$ and $k_{d2}$ represent an *association* and a *dissociation* rate constant between B and C, respectively.

For the calculation, we set initial values of the components, following the 3 h procedures of sandwich structure formation (sandwich structures including HA protein in **METHODS**). The initial concentration of A was 1.67×10^-8^ M. We chose the initial concentration of B in the range from 10 aM to 1 nM. The concentration of C remained 0 in the first 2 h of the formation. After 2 h from the beginning, 2×10^-11^ M was applied to [C] in the equations. The concentrations of all others were chosen as zero at the beginning. We set $k_{a1}$, $k_{d1}$, $k_{a2}$, and $k_{d2}$ as *10^4^*, *10^-4^*, *10^4^*, *10^-4^* (A.U.), respectively.

Then, for numerical analysis, we replace the differential rate equations with finite difference approximations, where Δt is the size of the time step (1 s).

$\frac{d[A]}{dt}\approx\frac{\Delta[A]}{\Delta t}$ (7)

$\frac{d[B]}{dt}\approx\frac{\Delta[B]}{\Delta t}$ (8)

$\frac{d[C]}{dt}\approx\frac{\Delta[C]}{\Delta t}$ (9)

$\frac{d[AB]}{dt}\approx\frac{\Delta[AB]}{\Delta t}$ (10)

$\frac{d[BC]}{dt}\approx\frac{\Delta[BC]}{\Delta t}$ (11)

$\frac{d[ABC]}{dt}\approx\frac{\Delta[ABC]}{\Delta t}$ (12)

The concentration of each component at n^th^ time step (t_n_) was calculated with following equations.

$\left[ A\left( t_{n} \right) \right]=\left[ A\left( t_{n-1} \right) \right]+\frac{\Delta[A]}{\Delta t}{(t}_{n-1})\Delta t$ (13)

$\left[ B\left( t_{n} \right) \right]=\left[ B\left( t_{n-1} \right) \right]+\frac{\Delta[B]}{\Delta t}{(t}_{n-1})\Delta t$ (14)

$\left[ C\left( t_{n} \right) \right]=\left[ C\left( t_{n-1} \right) \right]+\frac{\Delta[C]}{\Delta t}{(t}_{n-1})\Delta t$ (15)

$\left[ AB\left( t_{n} \right) \right]=\left[ AB\left( t_{n-1} \right) \right]+\frac{\Delta[AB]}{\Delta t}{(t}_{n-1})\Delta t$ (16)

$\left[ BC\left( t_{n} \right) \right]=\left[ BC\left( t_{n-1} \right) \right]+\frac{\Delta[BC]}{\Delta t}{(t}_{n-1})\Delta t$ (17)

$\left[ ABC\left( t_{n} \right) \right]=\left[ ABC\left( t_{n-1} \right) \right]+\frac{\Delta[ABC]}{\Delta t}{(t}_{n-1})\Delta t$ (18)

Firstly, the concentrations of all components at 1 s (t_1_) were calculated with the initial condition. Then, the calculated values at t_n-1_ were used to calculate values at the next time step t_n_. Finally, we obtained the final concentrations of ABC, at t_10800_. We repeated calculations with the different initial concentrations of B. The normal concentrations of ABC were calculated by normalizing the final concentrations of ABC with respect to their maximal values at the high concentration condition of B.

The simulation results show that, at a rather low target concentration, the number of sandwich structures is linearly proportional to that of target concentrations. Since our sensor signal is proportional to the number of sandwich structures, we can expect a linear response curve for our sensors.

**Fig. S3**


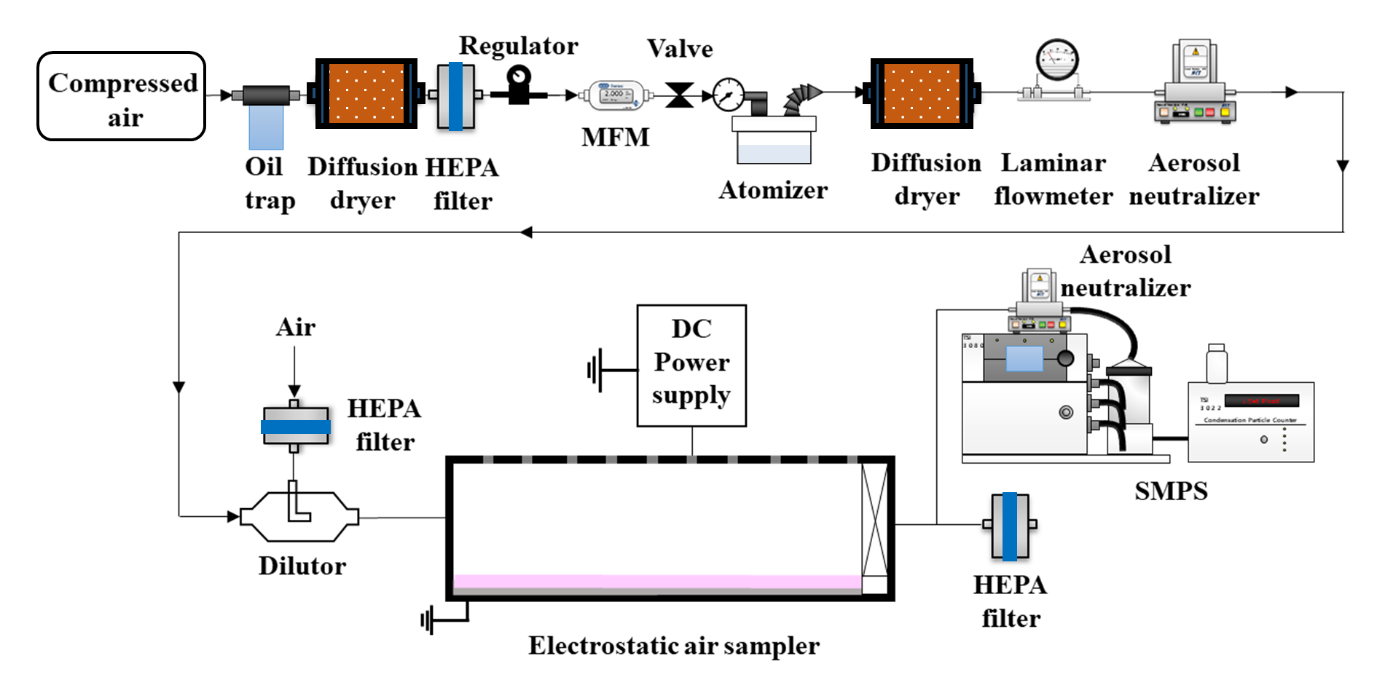


**Fig. S3** Experimental set-up for the aerosolization and collection of viruses.

**Fig. S4**


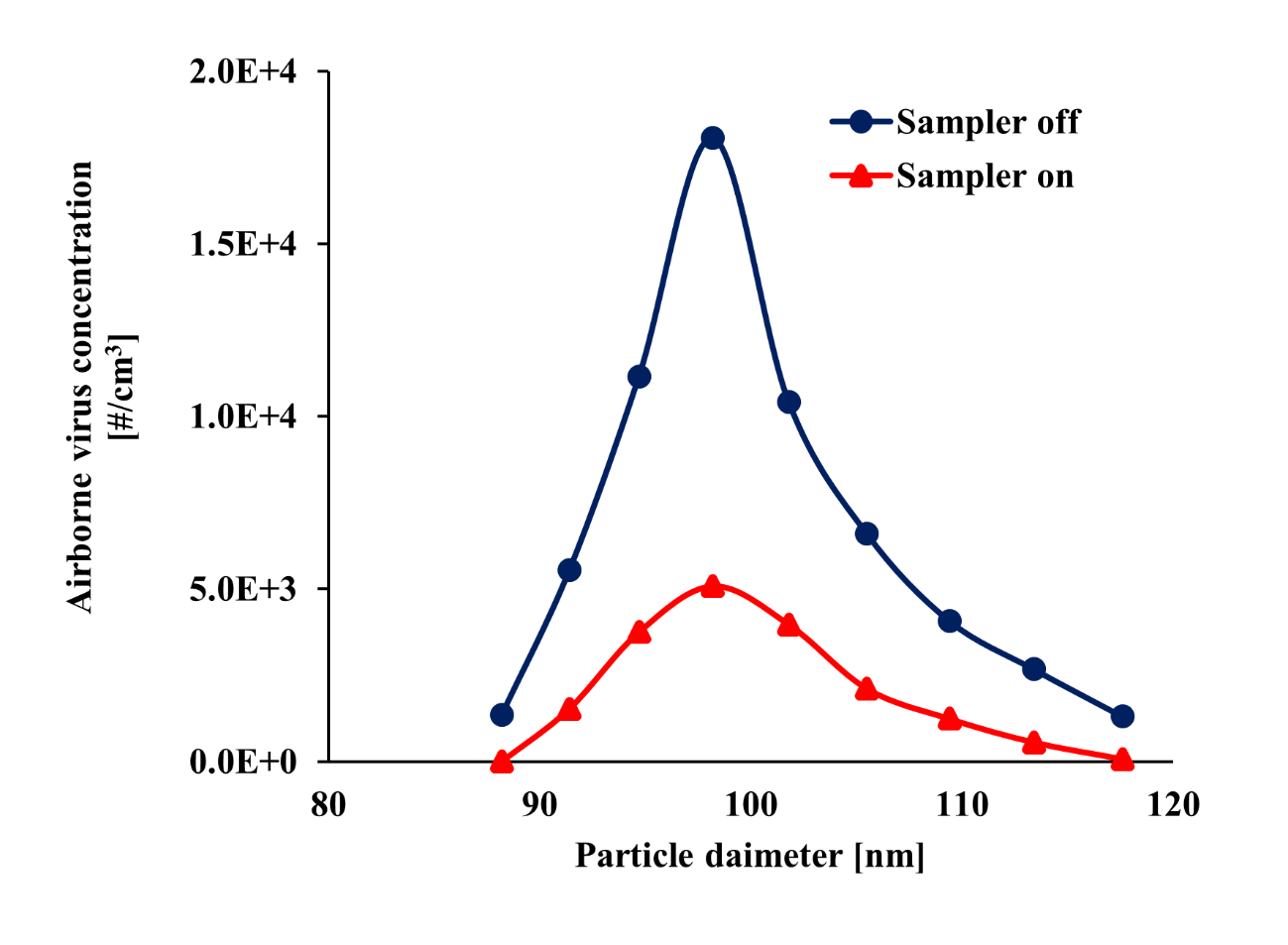


**Fig. S4** shows the number concentration of aerosolized virus measured when the air sampler was *off* and *on*. The peak diameter of the aerosolized virus was 97 nm which is similar to previously reported studies^1, 2^. The virus collection efficiency of the sampler was 0.7 ± 0.04 with a sampling flow rate of 100 L/min and the applied voltage of -10 kV.

**METHODS**

**Preparation of influenza A (H1N1) virus solution.** Influenza A virus solutions with 4.33 × 10^6^ PFU/mL were donated by BioNano Health Guard Research Center (H-GUARD). The virus solution was aliquoted in 1 mL and stored at -70 ℃. To determine the number of plaques by influenza A virus, plaque assays were carried out on just confluent monolayers of MDCK cells in 6-well plates. Serial dilutions of the initial stock viruses were carried out with MEM. The growth medium was removed from the 6-well plates and each diluted virus was added into wells in duplicate. The plates were incubated for 1 h at 37 ℃ and unadsorbed viruses were aspirated off and then overlaid with MEM containing 0.6 % agarose gels. After incubation for 3 days at 37 ℃ under 5 % CO_2_, the plates were stained with crystal violet. The plaque was counted by microscope observation. Virus titers were calculated by the Lorenz and Bogel calculation method^3^.

**Sandwich ELISA test procedures with HA** *HRP conjugated* *1^st^ antibody* and *2^nd^ antibody* were prepared as *detection* and *capture* antibody respectively. A 96-well polystyrene microplate (Corning, Inc., USA) was coated with 100 µL of capture antibody solution. The plate was sealed and incubated overnight at 4 ℃. Each well was washed 3 times with 250 µL of wash buffer (PBS with 0.05 % Tween 20). Wells were blocked with 200 µL of ELISA/ELISPOT diluent (1×, Thermo Fisher Scientific). The plate was incubated for 1hour at room temperature. The wells were washed 1 time with 250 µL of wash buffer. 100 µL of HA solution with different concentrations was introduced to each well. After sealing the plate, we incubated it for 2 hours at room temperature. Then, each well was washed 5 times with 250 µL of wash buffer. 100 µL of detection antibody solution was added to the wells. After the incubation of the plate for 1 hour at room temperature, each well was washed as previously. 100 µL of TMB solution (Thermo Fisher Scientific) was mixed into each well. The mixed solutions were incubated for 15 minutes at room temperature. 50 µL of stop solution (1 M H_3_PO_4_) was added to each well. The absorbance of the assay solution was obtained using a microplate reader at 450 nm.

**REFERENCES**

1. Quan FS, Rubino I, Lee SH, Koch B, Choi HJ. Universal and reusable virus deactivation system for respiratory protection. *Sci Rep-Uk* **7**, 39956 (2017).

2. Hagbom M, Nordgren J, Nybom R, Hedlund KO, Wigzell H, Svensson L. Ionizing air affects influenza virus infectivity and prevents airborne-transmission. *Sci Rep-Uk* **5**, 11431 (2015).

3. L.J. REED HM. A SIMPLE METHOD OF ESTIMATING FIFTY PER CENT ENDPOINTS. American Journal of Epidemiology 27, 493–497 (1938).
